# Wound healing activities of different extracts of *Centella asiatica* in incision and burn wound models: an experimental animal study

**DOI:** 10.1186/1472-6882-12-103

**Published:** 2012-07-20

**Authors:** Juraiporn Somboonwong, Mattana Kankaisre, Boonyong Tantisira, Mayuree H Tantisira

**Affiliations:** 1 Department of Physiology, Faculty of Medicine, Chulalongkorn University, Patumwan, Bangkok, 10330, Thailand; 2 Inter-disciplinary Program of Pharmacology, Graduate School, Chulalongkorn University, Patumwan, Bangkok, 10330, Thailand; 3 Department of Pharmacology and Physiology, Faculty of Pharmaceutical Sciences, Chulalongkorn University, Patumwan, Bangkok, 10330, Thailand; 4 Present address: Faculty of Pharmaceutical Sciences, Burapha University, Chonburi, 20131, Thailand

**Keywords:** Centella asiatica, Wound healing, Incision wound, Burn wound, Asiatic acid, β-sitosterol, Asiaticoside, Madecassocide

## Abstract

**Background:**

The efficacy of *Centella asiatica* for incision and burn wounds are not fully understood. Here, we report the wound healing activities of sequential hexane, ethyl acetate, methanol, and water extracts of *Centella asiatica* in incision and partial-thickness burn wound models in rats.

**Methods:**

Male Sprague–Dawley rats weighing 250–300 g were randomly divided into incision and burn wound groups. Each group was stratified into seven subgroups: (1) untreated; (2) NSS-; (3) Tween 20**®**- (vehicle control); (4) hexane extract-; (5) ethyl acetate extract-; (6) methanol extract-; and (7) aqueous extract-treated groups. The test substances were applied topically once daily. The tensile strength of the incision wound was measured on the seventh day after wound infliction. The general appearance and degree of wound healing of the burn wound were assessed on Days 3, 7, 10 and 14 after burn injury and prior to histopathological evaluation.

**Results:**

On the seventh day after wound infliction, the tensile strength of incision wound in all extract-treated groups was significantly higher than that of the vehicle control (Tween 20®), but comparable to the NSS-treated group. The degrees of healing in the burn wound with the four extracts were significantly higher than that of the control on Days 3, 10 and 14. Histopathological findings on Day 14 after burn injury revealed prominent fibrinoid necrosis and incomplete epithelialization in the control and untreated groups, whereas fully developed epithelialization and keratinization were observed in all extract-treated groups. Analysis by thin layer chromatography demonstrated that the phyto-constituents β-sitosterol, asiatic acid, and asiaticoside and madecassocide were present in the hexane, ethyl acetate and methanol extracts, respectively.

**Conclusions:**

All extracts of *Centella asiatica* facilitate the wound healing process in both incision and burn wounds. Asiatic acid in the ethyl acetate extract seemed to be the most active component for healing the wound.

## Background

A wound is an injury to a part of the body, especially one in which a break is made in the skin. There are various types of wounds, including an incised wound, lacerated wound, abrasion, contusion, ulcer, and burn wound [1]. Treatment of the wound usually involves preventing infection because the skin, the body’s barrier to infection, is destroyed. Burn wounds require treatment according to the severity of the burn. Minor burns are generally treated with topical ointment and dressing, while severe burns need immediate medical attention and hospitalization [2]. Inappropriate caring of the wound may delay its healing, causing the area to become infected and subsequently resulting in chronic wounds. Antimicrobial ointments such as silver sulfadiazine, mafenide, silver nitrate, povidone-iodine, mupirocin and bacitracin, are used to reduce the risk of infection in minor cuts and burns. However, these topical antimicrobials have some side effects and are only partially effective in healing the wound [3]. Hence, there is a need for newer drugs to heal the wound.

Development of drugs or agents to treat wounds has been performed for many years. Currently, several medicinal plants have been integrated into the health care system, especially the primary healthcare system [4]. One of the plants studied is the *Centella asiatica* (Linn.) Urban. This plant is commonly found in many parts of the world, including Asia and the Middle East [5]. In Asia, *C. asiatica* has long been used in traditional medicine because of its ability to heal wounds, improve mental clarity, and treat skin conditions such as leprosy and psoriasis [6]. The therapeutic substances in *C. asiatica* are saponin-containing triterpene acids and their sugar esters, of which asiatic acid, madecassic acid and asiaticosides are considered to be the most important [7].

It has been reported that 1% *C. asiatica* extract cream improves wound healing of chronic ulcer in width, length and depth after 7, 14 and 21 days of use of the product [8]. In one study of the effect of *C. asiatica* extract on acute radiation dermatitis in rats, the wounds in the treatment group were less severe and the repair processes began earlier than those in the control group [9]. A study in an incision wound model demonstrated that the ethanolic extract of *C. asiatica* significantly increased the wound breaking strength [10]. Phyto-constituents present in *C. asiatica* have been found to be responsible for these wound healing properties. Asiaticoside isolated from *C. asiatica* increased hydroxyproline content, tensile strength, collagen content and epithelialization in a punch wound model [11]. Furthermore, triterpenes from *C. asiatica* were shown to increase remodeling of the collagen matrix and stimulate glycosaminoglycan synthesis in a rat wound chamber model [12]. Oral administration of madecassoside (6, 12, 24 mg/kg) isolated from *C. asiatica* herbs facilitated burn wound healing in mice through its antioxidative activity, collagen synthesis and angiogenesis [13]. In addition, an oral *C. asiatica* extract capsule has been proved to be effective in promotion of wound healing and scar suppression in patients with diabetes-related wounds, with no serious side effects [14].

Few studies have investigated the efficacy of *C. asiatica* on incision and burn wounds, which have different healing mechanisms compared to excision wounds. Thus, the purpose of this study was to evaluate the wound healing activities of sequential hexane, ethyl acetate, methanol, and aqueous extracts of *C. asiatica* in incision and partial-thickness burn wound models in rats.

## Methods

### Preparation and extraction of *C. asiatica*

Collected aerial parts of *C. asiatica* were oven-dried at 50°C and then powdered using a milling machine. The powdered plant (1 kg) was macerated with *n-*hexane (3 × 4 L, 3 days each) at room temperature. The pooled filtrates were dried under reduced pressure to give the *n*-hexane extract (HexE) at a constant weight of 17.8 g (0.18% yield of fresh plant). The marc was air-dried before it was further macerated with ethyl acetate and methanol, respectively, using the procedure described above to give the ethyl acetate (EtAcE, 32.7 g, 0.33% yield) and methanol (MeE, 267.5 g, 2.68% yield) extracts. Subsequently, a portion (300 g) of the dried marc was boiled in distilled water (2 L) for 3 h, then filtered and dried to give 95.1 g of the hot aqueous extract (AqE, 3.17% yield). All solvents used were of commercial grade and were obtained from U & V Holding Ltd., Bangkok, Thailand.

Tween 20® (polyoxyethylene (20) sorbitan monolaurate) as a 10% solution in distilled water was used as vehicle for preparation of a 10% (w/v) of each extract for topical application. Ten grams of the dried extract was triturated with 9 mL of vehicle. Vehicle was then added gradually until the final concentration of the extract was 10%.

### Thin layer chromatography (TLC) profiling of the extracts

Characterization of the extracts was performed by TLC with a silica gel 60 F_254_ plate. The solvent systems used for analysis of HexE, EtAcE, MeE, and AqE were as follows: chloroform: acetate (9:1), chloroform: methanol (9:1), ethyl acetate: methanol: water (4:0.4:0.2) and ethyl acetate: methanol: water (4:0.4:0.3), respectively. The TLC plate for each extract was sprayed with 10% sulfuric acid in ethanol and then heated on a hot plate at a constant temperature of 100°C for 15 min.

### Experimental protocol

A total of 112 male Sprague–Dawley rats weighing 250–300 g obtained from the National Laboratory Animal Center, Mahidol University, Nakornpathom, Thailand were used in this study. The rats were caged in special rooms with a temperature of 25 ± 1°C, free access to commercial pellet diet and water *ad libitum*. The rats were used 7 days after arrival in the animal facility. This 7-day period allowed the animals to acclimatize to the laboratory environment. Animal experiments in this study were carried out in accordance with the ARRIVE (Animals in Research: Reporting *In Vivo* Experiments) guidelines [15] and the Guide for the Care and Use of Laboratory Animals of the National Research Council of Thailand. All care was taken to minimise the suffering of the animals. The experimental protocol was approved by the Ethics Committee of the Faculty of Pharmaceutical Sciences, Chulalongkorn University.

The animals were randomly divided into incision and burn wound groups with each group having 56 animals. Each group was further divided into seven subgroups composed of eight animals per subgroup: 1) untreated group, 2) NSS-treated group, 3) Tween 20®-treated (vehicle control) group, 4) HexE-treated group, 5) EtAcE-treated group, 6) MeE-treated group, and 7) AqE-treated group.

### Effect of *C. asiatica* extracts on healing of incision wounds

The effect of *Centella asiatica* extracts on the healing of incision wounds was investigated by using the model of Baie and Shiekh [16]. The animals were anesthetized intraperitoneally with sodium pentobarbital at 60 mg/kg BW during induction of the wound. The right side on the back of each animal was shaved and depilated. Next, a 3 cm long, midline incision was made through the skin with a sharp scalpel and was closed with a 0.5 cm spaced interrupted sutures with black silk no.3-0 to secure the edges. After creating the wound, 0.5 mL of the test substance was topically applied to the affected area once daily. On the seventh day after inflicting the wound, the animals were sacrificed with intra-peritoneal injection of sodium pentobarbital at 100 mg/kg BW. Sutures were removed and tissues were isolated from the healed wound to assess its tensile strength, as described below.

### Assessment of the tensile strength of incision wounds

Healing of incision wounds was evaluated by measuring the tensile strength on day 7 after inflicting the wound. Sutures were removed and the skin tissue was cut 1 cm away from each side of the wound. The isolated wound tissues were used to measure the load (force) required to break the tissue with a computerized tensiometer (EZ-TEST I 30804100798, Shimadzu Corp., Japan). Tensile strength was calculated using the following formula: tensile strength (N/cm^2^) = breaking force (N)/area (cm^2^); where area (cm^2^) = thickness (cm) × width (cm) [16].

### Effect of *C. asiatica* extracts on healing of burn wounds

The effect of *C. asiatica* extracts on the healing of the burn wounds was investigated using the method described by [Somboonwong et al.], which was modified from Zawacki [18]. The animals were anesthetized with intra-peritoneal injection of sodium pentobarbital at 60 mg/kg BW during induction of the wound. The back of the animal between the lower parts of both scapulas were shaved and depilated. Next, a partial thickness burn was made by putting a hot plate (3.5 × 4.6 cm) at a temperature of 75°C on the prepared area for 10 s. The burnt area was about 10% of the total body surface area. After burning, 0.5 ml of the test substances was topically applied to the burnt area once daily. The burnt area was measured immediately after the burn and on Days 3, 7, 10 and 14 after burn injury using millimeter-scale graph paper. The degree of wound healing was calculated as described below. On day 14 after burn injury, the animals were sacrificed with intra-peritoneal injection of sodium pentobarbital at 100 mg/kg BW. Tissue of the healed wound (0.5 × 0.5 cm) was collected from each animal for histological examination.

### Gross examination of the burn wound lesion

The wound was grossly examined on Days 3, 7, 10 and 14 after burn injury. The lesion of the wounds was examined using the following criteria: wound bed, color, exudates, swelling of the wound surface, and the consistency of tissues surrounding the wound.

### Assessment of the degree of healing of burn wounds

On Days 3, 7, 10 and 14 after burn injury, color photographs of the wounds were taken by digital camera. The areas of the wound were measured by tracing the wound boundaries using millimeter-scale transparent graph paper with a permanent marker. The degree of wound healing was calculated using the following formula: degree of wound healing (%) = 1-[wound area on the corresponding day (cm^2^)/wound area on day zero (cm^2^)] × 100 [19].

### Histopathological examination of burn wounds

A specimen of the skin (0.5 × 0.5 cm) was taken from the middle of the burnt area. The tissue was preserved in a 10% fresh, neutral buffered solution of formaldehyde for at least 24 h. Sections were stained with hematoxylin and eosin dyes and examined using a light microscope (Nikon 516609) with × 20 and × 40 objective lens.

### Statistical analysis

Results are presented as mean ± standard error of mean (SEM). Differences among experimental groups were compared by one-way analysis of variance (ANOVA), followed by the least significant different test (LSD), and were considered significant when *P* was less than 0.05.

## Results

### Characteristic features of *C. asiatica* extracts

The major active compounds in HexE, EtAcE and MeE were identified by TLC as β-sitosterol, asiatic acid, and asiaticoside and madecassoside, respectively. None of these components were found in AqE.

### Effect of *C. asiatica* extracts on healing of incision wounds

As shown in Table 1, the tensile strengths of untreated and Tween 20®-treated wounds were 14.99 ± 1.72 and 14.69 ± 0.72 N/cm^2^, respectively. A significant increase of tensile strength was observed in all extract-treated groups (21.20 ± 2.24, 21.26 ± 1.77, 19.72 ± 0.84 and 21.05 ± 1.90 N/cm^2^ for HexE, EtAcE, MeE and AqE, respectively) compared to the vehicle-treated group. However, none of the tensile strengths in the extract-treated groups differed significantly from that of the NSS-treated group.

**Table 1:** **Tensile strength of incision wounds treated with extracts of *Centella asiatica *on day 7 after wound infliction**

**Treatment group**	**Tensile strength (N/cm**^**2**^**)**
Untreated	14.99 ± 1.72
NSS	17.62 ± 1.42
Tween 20®	14.69 ± 0.72
Hexane extract	21.20 ± 2.24^**^
Ethyl acetate extract	21.26 ± 1.77^**^
Methanol extract	19.72 ± 0.84^*^
Aqueous extract	21.05 ± 1.90^**^

Values are presented as mean ± S.E.M; n = 8; ^*^P<0.05 and ^**^P<0.01 vs. Tween 20®.

### Effect of *C. asiatica* extracts on healing of burn wounds

#### Burn wound lesions

On Day 3, the wound in the untreated, NSS-treated and Tween 20®-treated groups became swollen and bruised. In contrast, the wounds in all extract-treated groups showed a mild degree of swelling and the wound surface was rather dry. Most wounds in the EtAcE-treated group had begun to contract from the wound edge.

On Day 7, all wounds in the untreated, NSS-treated and Tween 20®-treated groups were dark red, showed thickening of the skin at the wound site, and remained unchanged in size from the first day. Most of the wounds treated with the extracts showed wound contraction compared with the control group. The wounds in the MeE-treated group showed marked hair growth. Some wounds in the MeE- and AqE-treated groups had scabs covering the wound surface.

On Day 10, all wounds in the untreated, NSS-treated and Tween 20®-treated groups showed moderate exudation and no hair growth, with scabs covering the wound surface. All wounds in the extract-treated groups had a dry surface, progressive wound contraction, and increased hair growth.

On Day 14, the last day of the experiment, all wounds in the untreated, NSS-treated and Tween 20®-treated groups showed moderate exudation and scabs starting to separate from the wound surface. The wounds in the extract-treated groups showed a marked reduction in size and continuous growth of hair at the wound site. The wounds in the MeE- and AqE-treated groups showed the most marked reduction in wound size.

### Degree of healing of burn wounds

The effects of *C. asiatica* extracts on the degree of healing of burn wounds are shown in Table 2. The degree of wound healing did not differ significantly among the untreated, NSS-treated and Tween 20®-treated groups at every time point. On Day 3 after burn injury, only animals from the EtAcE-treated group had a higher degree of wound healing compared to the vehicle control group. Similar results were observed on Day 7.

**Table 2:** **Degree of healing of partial-thickness burn wounds treated with extracts of *Centella asiatica***

**Treatment group**	**Degree of wound healing (%)**			
	**Day 3**	**Day 7**	**Day 10**	**Day 14**
Untreated	9.51 ± 1.21	14.27 ± 2.10	20.21 ± 2.10	25.36 ± 1.81
NSS	12.80 ± 2.65	19.64 ± 2.34	25.90 ±2.67	31.85 ± 2.66
Tween 20®	10.05 ± 1.50	20.57 ± 2.58	27.69 ± 2.39	38.07 ± 5.15
Hexane extract	11.12 ± 2.89	26.02 ± 4.02	37.78 ± 3.89^*^	53.87 ± 4.64^*^
Ethyl acetate extract	18.13 ± 2.29^*^	30.17 ± 1.81^*^	38.11 ± 2.30^*^	57.53 ± 5.68^*^
Methanol extract	11.71 ± 1.94	20.39 ± 2.07	28.94 ± 2.55	60.31 ± 5.70^**^
Aqueous extract	7.86 ± 1.45	19.98 ± 2.23	28.57 ± 3.35	59.82 ± 8.31^**^

Values are presented as mean ± S.E.M; n = 8; ^*^P < 0.05 and ^**^P < 0.01 vs. Tween 20®.

On Day 10 after burn injury, the degree of wound healing in animals treated with HexE or EtAcE was significantly higher than that in the vehicle control group. Moreover, the degree of wound healing in the HexE-treated group was not significantly different from that in the EtAcE-treated group.

On Day 14 after burn injury, the degree of wound healing in animals in all extract-treated groups was significantly higher than that in the vehicle control group. The degree of wound healing did not differ significantly among the extract-treated groups.

### Histopathological evaluation

**Figure 1:**
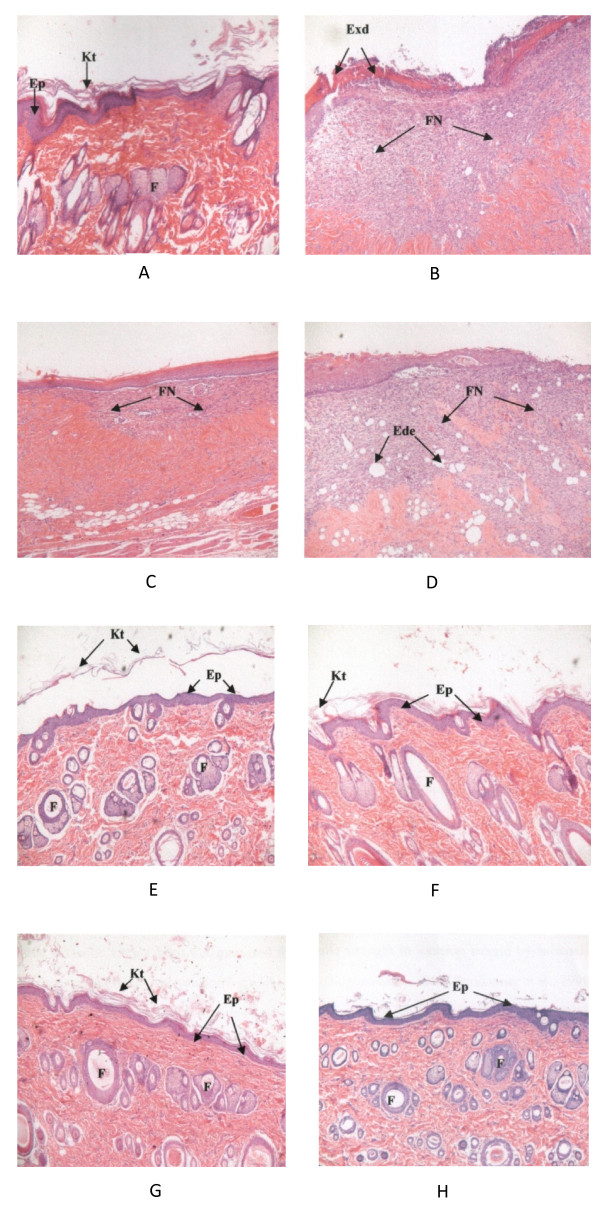
**Histological features of partial-thickness burn wounds on day 14 after burn injury. **Hematoxylin and eosin stains of **A**) normal skin; **B**) untreated wound; **C**) NSS-treated wound; **D**) Tween 20-treated wound; **E**) hexane extract-treated wound; **F**) ethyl acetate extract-treated wound; **G**) methanol extract-treated wound; **H**) water extract-treated wound. Ep: epidermis; Kt: keratin; Exd: exudates; FN: fibrinoid necrosis; Ede: edema; F: hair follicle.

Histological features of normal skin are illustrated in Figure 1A. At the end of the experiment, on Day 14 after burn injury, the untreated burn wound showed prominent fibrinoid necrosis in the subepidermal region, which was characterized by permeation of collagen with fibrin and additional degenerative changes. Re-epithelialization was incomplete with little skin appendages. The wound surface was covered with exudates (Figure 1B).

The wounds in the NSS- and Tween 20®-treated groups showed fibrinoid necrosis in some areas of the subepidermis. There were empty spaces in the dermal region of the Tween 20®-treated group, indicating evidence of edema. Re-epithelialization was incomplete, but the wounds in the NSS-treated group looked better than those in the Tween 20® -treated group (Figures 1C and 1D, respectively). The wounds in all extract-treated groups showed fully developed epithelialization and keratinization. Skin appendages were almost normal. There was no noticeable necrosis or inflammation (Figures 1E, 1F, 1G and 1H).

## Discussion

This study demonstrated that four different extracts of *C. asiatica* were able to increase the rate of wound healing for both incision and burn wounds. Seven days after inflicting the wound, the tensile strengths in the HexE, EtAcE, MeE and AqE-treated groups were significantly higher than that in the vehicle-treated group, but similar to the NSS-treated group. This observation may be due to the incision wound having minimal cell loss, since in this situation a tissue injury can be closed within 6–12 h if not contaminated [1]. Additionally, NSS, the most commonly used solution in clinical practice for wound irrigation, preserves the physiological condition and does not contain surfactants such as Tween 20®, which has been postulated to cause skin injury [20]. Also, it is well known that collagen is the major protein component of wound connective tissue and is responsible for the tissue strength [21]; thus, the increased tensile strengths observed as a characteristic of the healing activity of extracts of *C. asiatica* may indicate an increase in collagen in the wound lesion. The present findings agree well with previous studies showing an increase of tensile strength or collagen synthesis in wounds treated by ethanolic extracts [22,23], asiaticoside [11,24] or triterpenoids isolated from *C. asiatica*[12,25]*.*

In burn wounds, there is an extensive loss of cells and tissues compared to an incision wound, and this makes the repair process more complicated [26]. Wound healing activities of HexE, EtAcE, MeE and AqE were also observed, but at different time points. Animals treated with EtAcE, HexE, MeE and AqE had a significantly higher degree of healing on Days 3, 10 and 14 compared to the vehicle-treated group. However, on Day 14, there was a similar degree of wound healing in all the groups. All extract-treated wounds appeared to heal better than the controls based on gross examination, degree of wound healing, and histopathological evaluation.

Differences in the major constituents of the extracts may account for the results of the study. Asiatic acid, which was identified mainly in EtAcE, is the most potent component of *C. asiatica* for induction of expression of TNFAIP6, a hyaladherin involved in extracellular matrix remodeling and modulation of inflammation, in human fibroblasts [27]. Asiatic acid has also been found to increase collagen synthesis, which is important in the healing process [12,25]. These observations may explain the rapid onset on Day 3 and the sustained positive effects of EtAcE on burn wounds. The phases of inflammation and proliferation gradually increased between Days 7 and 10.

Significant effects of HexE appeared later, on Day 10 and Day 14. TLC showed that the major constituent of HexE was β-sitosterol, which has a potent angiogenic activity [28,29]. Thus, the healing activity of HexE observed in gross examination and histopathology could be partly due to angiogenic activity. The healing effects of MeE and AqE were significant on Day 14, indicating that the major constituents of these two extracts might exert their effect on the late phase of wound healing. Asiaticoside and madecassocide are triterpenoids found in MeE. Asiaticoside induces type I collagen synthesis in human dermal fibroblast cells via activation of the TGFbeta receptor I kinase-independent Smad signaling pathway [30] and also elevates antioxidant levels in a punch wound model, thereby promoting wound healing [31]. Madecassoside has also been shown to facilitate burn wound healing in mice [13].

β-sitosterol, asiatic acid, asiaticoside and madecassocide were not found in AqE using TLC but wound healing activity of this extract was seen on Day 14. Anand *et al.* found a high flavonoid content in an aqueous extract of *C. asiatica* and these molecules possess anti-oxidant property [32]. Antioxidants may help to control wound oxidative stress and thereby accelerate wound healing [33]. Therefore, flavonoids in the aqueous extract may have played an important role as anti-oxidants in the late stage of wound healing. These molecules could not be determined by the TLC technique used in the study. TLC is the most widely used method to detect substances because of its simplicity, but one of its limitations is its inability to detect certain molecules.

The results of this study support the notion that *C. asiatica* can promote wound healing by inhibiting inflammation, inducing collagen synthesis, promoting angiogenesis, inducing vasodilation, and reducing wound oxidative stress. In addition, *C. asiatica* extracts have been shown to affect cellular growth and proliferation in injured tissues. In an ideal wound healing situation, new tissue growth replaces damaged tissue causing functional or cosmetic impairment. The wound healing activity of *C. asiatica* extracts may be related to the growth factors such as endothelial growth factor, fibroblast growth factor and vascular endothelial growth factor. A further study on the effects of *C. asiatica* extracts on these growth factors is needed to clarify the mechanism of wound healing. Microcirculatory studies are also warranted to investigate the anti-inflammatory activity of EtAcE of *C. asiatica,* which was seen early during the healing process.

## Conclusions

The present study demonstrates the wound healing effects of *C. asiatica* extracts for both incision and burn wounds. All types of extracts (HexE, EtAcE, MeE and AqE) exerted wound healing activity, but EtAcE containing asiatic acid was the most active extract for wound healing.

## Abbreviations

C. asiatica: Centella asiatica; NSS: Normal saline solution; TLC: Thin-layer chromatography; HexE: Hexane extract; EtAcE: Ethyl acetate extract; MetE: Methanol extract; AqE: Aqueous extract.

## Competing interests

All authors declare that they have no competing interests.

## Authors’ contributions

JS participated in the design of the experiments, conducted and analyzed the gross and histopathological data, and drafted the manuscript. MK was responsible for preparing incision and burn wounds and for statistical analysis. BT prepared the extracts and acquired animal data. MT conceived and designed the study, analyzed the data and critically revised the manuscript. All authors have read and approved the final manuscript.

## Pre-publication history

The pre-publication history for this paper can be accessed here:

http://www.biomedcentral.com/1472-6882/12/103/prepub
